# Analysis of Requirements for Developing an mHealth-Based Health Management Platform

**DOI:** 10.2196/mhealth.5890

**Published:** 2017-08-03

**Authors:** Hehua Zhang, Han Zhang, Xiaoning Wang, Zuosen Yang, Yuhong Zhao

**Affiliations:** ^1^ Shengjing Hospital of China Medical University China Medical University Shenyang, Liaoning China; ^2^ Department of Medical Informatics China Medical University Shenyang, Liaoning China; ^3^ Health Information Center of Liaoning Province Shenyang, Liaoning China; ^4^ Shengjing Hospital of China Medical University China Medical University Liaoning, Shenyang China

**Keywords:** online information-seeking, health management, mHealth

## Abstract

**Background:**

Studies have consistently shown that mobile and Web-based apps have positive impacts on people’s daily lifestyles, health management, and disease treatment. As the development of medical and health informatization in China has evolved, different kinds of mobile-based apps for individuals and hospitals have been developed by software vendors. However, doubts and challenges posed by the media have prevented these apps from having a stable and substantial user base. Analyses of user requirements have not typically been performed prior to the design of such mobile apps. The health information government authority in Liaoning Province, China, was planning to establish a mobile health (mHealth)-based health management platform, aiming to alleviate the difficulties citizens have in seeking hospital services.

**Objective:**

The goal of this study was to determine the actual health and medical needs of citizens that may be addressed by medical information technologies. The results may contribute to the functional design and development of health management and appointed treatment-oriented mobile apps.

**Methods:**

In this study, a semi-structured questionnaire on mHealth requirements was designed and tested, and 240 questionnaires were given to the outpatients of the First Hospital of the China Medical University in Shenyang, Liaoning Province, China; of these, 228 valid responses were collected, for a response rate of 95%. We discussed the current development of mHealth with 50 related experts and engineers from health authorities and a medical information company. SPSS 13.0 was used for statistical analyses.

**Results:**

After detailed analyses of the questionnaire data, several findings were evident: first, most citizens and patients were unclear about their health conditions (64.5%, 147/228) and were interested in receiving a mobile app as a tool to manage their health and medical needs (71.1%, 162/228). Patients in different outpatient departments had different opinions regarding online registration. Conversely, the main problems for outpatients were long waiting times (66.4%, 148/223) and difficulties in making appointments (46.5%, 106/228), and they also worried about payments and Internet problems when using a mobile app for appointment reservations. Furthermore, as the main service target of mHealth is the health management of the general population, we first need to solve the associated interoperability and data security problems associated with such apps.

**Conclusions:**

This study provides insight into the health and medical requirements of smartphone apps, and draws attention to some of the challenges and opportunities of mHealth. We suggest several value-added features and characteristics that app developers should take into consideration when developing health and medical-related apps. The findings also highlight some major challenges that require further consideration and research to ensure that these apps meet the core needs of patients and aid the development of the health information system in Liaoning Province, China.

## Introduction

### Development and App of mHealth Apps

The concept of mobile health (mHealth) appeared in the early 21st century with the development of mobile phones and wireless technology [1], and since that time research has consistently shown the deployment of mHealth will benefit patients with chronic disease [2], making it easier to implement primary health care [3] and reduce health care costs [4]. Different kinds of mobile phone or tablet-based apps on Android and/or iOS operating systems have been exploited for use inside [5] and outside of hospitals for the purposes of nursing support, outpatient management, chronic disease management, online medical consulting, daily health, and public service [4,6,7]. Research on evidence-based practices consistently show the positive effects of using mHealth-based apps [8-10]. In China, mHealth is in a stage of rapid growth, and many mHealth apps that are not specifically used by health care providers are familiar to (and used by) patients outside of hospitals, including online doctor services that are available at any time, health data tracking, and real-time analyses using wearable devices and self-help checking. Some hospitals have even developed their own hospital mobile apps in cooperation with online payment companies. Governments in China are taking positive measures to encourage mHealth development, and venture capital funds are investing in mHealth apps to promote the prosperity of the health market [11].

### mHealth-Based Health Management Platform in Liaoning Province, China

The entire health information structure of Liaoning Province is at the forefront of development in China. The system includes five parts known as *46312*: a four-level health information platform integrating county-city-province to nation; six key business apps (medications, medical service, health care, health insurance, fertility service, and integrated apps); three-database construction (electronic medical records, electronic health records, and population); one virtual private network connecting all platforms and apps; and a two-standard hierarchy (health information and security). The ultimate aim of this structure is to provide more convenient services for patients using current information technologies, which is the underlying reason for genesis of this project involving an mHealth-based health management platform in Liaoning Province (referred to as *the platform* in the sections below). To alleviate the difficulties that regional citizens experience while seeking medical and health services [12], the platform will be integrated with large regional hospitals by adopting data exchange and mHealth-related technologies, which would solve the main problems that patients confront by providing multiple ways of accessing medical and health services (including mobile phone apps, websites, and Wireless App Protocol-based apps) [13].

### Purpose of This Study

Various kinds of mHealth-based apps for patients are presently available on the Internet, yet none can maintain a stable and large-scale user base to support a range of province-based health requirements. We believe the underlying reason for this problem lies in the fact that existing apps do not meet the fundamental health requirements of the general public [14]. Compared with foreign research (eg, on online information-seeking, appointment making, and patient-provider interactions [15,16]), we have not found any comprehensive requirement studies of patients or experts on mHealth for the purpose of a system designed in China, except for some small Internet surveys that are not credible for platform development [17]. In this study, we aimed to acquire and analyze patients’ requirements of medical, health, and related mHealth apps in Liaoning Province, which will become the foundation for the functional design and development of a province-wide innovation platform in China.

## Methods

### Questionnaire Development

There were few preexisting questionnaires that were suitable for the investigation of mHealth requirements in this study, so we designed a new questionnaire that includes two parts: *Part A* for citizens and patients (30 questions), and *Part B* for health information-related persons (10 questions). Part A is divided into three sections: basic information questions, medical and health-related questions, and smartphone app questions (see the detailed contents in Multimedia Appendix 1). Part B concerns the current and future development (and current problems) of mHealth, as relayed by health information-related professionals. We invited four experts (three from the Medical Informatics Department of China Medical University and one from the Ministry of Health in Liaoning Province) to review the content and structure validity of the questionnaire. Subsequently, we distributed the questionnaire to 300 volunteers and, using SPSS13.0 to collect the data and assess its reliability, modified the questionnaire to make it workable for a large-scale investigation. The final reliabilities were 0.802 and 0.848 for Parts A and B, respectively.

### Participants

For questionnaire Part A, we randomly selected patients and related citizens (consisting of family or friends accompanying the patients) in six outpatient departments (medicine, dermatology, gynecology, ophthalmology, otorhinolaryngology, and mental health) of the First Hospital of China Medical University, which is the largest comprehensive hospital in Northern China with an outpatient capacity of over 2,630,000 per year. Patients with different kinds of disease conditions come from different cities in Liaoning Province because they prefer to visit this hospital first. Thus, the opinions of participants from the hospital outpatient areas represent a substantial proportion of the viewpoints of citizens in Liaoning Province. For Part B, we randomly chose engineers, clerks, and managers from three information centers in a hospital, Liaoning Province Ministry of Health, and an information technology (IT) company to complete the questionnaire.

### Survey Deployment

Three investigators visited the waiting areas of the six outpatient departments during the periods of December 2-4 and December 9-11 in 2013, when outpatient visits are typically at the highest level. Investigators individually explained the questionnaire to participants and asked the patients or citizens to fill it in, and retrieved each completed questionnaire. For Part B, we investigated health-related persons from three institutions that are the main participants in the health informatization process (a hospital information center, an information center of the health board, and an IT company). At the beginning of each questionnaire, we explained that the survey was part of a platform project that was proposed by China Medical University and that the information collected from the participants was only for research purposes. We did not give out the questionnaires until the patients understood this and were willing to complete the survey.

### Analysis

We used SPSS13.0 to process and analyze the questionnaire data, which included the following three parts: *Define Variables*, *Data Entry*, and *Data Statistics*.

#### Define Variables

In the variable view, each question item of the questionnaire was treated as a variable; we defined the name, data type, width, label, values, and other variables for each.

#### Data Entry

The types of questions were choice questions or multiple-choice questions; we entered all options selected by our respondents in the questionnaires into SPSS according to the defined variables.

#### Data Statistics

After data entry, we used the frequency, crosstabs, and tables in the descriptions function, defined multiple response sets, and conducted multiple response analyses. Cross tab analysis is a Chi-square test method to measure relevance between every two variables in SPSS13.0. Multiple response analysis is a frequency analyzed when there can be more than one response to a survey question per participant, and allows the set of responses to be combined and collectively analyzed.

## Results

### Overview

Two hundred and forty patients and citizens responded to questionnaire A, of whom 12 were excluded due to incomplete answers (more than two missing answers). At total of 228 valid respondents were included in our analysis (see Table 1). Many studies have pointed out that age and sex heavily affect online information-seeking behavior. We hypothesized that visit type (whether a person is visiting for the first time [first] or second time [subsequent], or is a companion [citizen] with patients visiting a hospital during the past 2 months) was a key factor for patients acquiring health information. Patients tend to go to large hospitals instead of general practitioners to seek medical treatment once they get sick. In the current health system of China, the more times that patients go to a hospital, the more they learn about their health conditions. Of the 228 respondents, almost half were women (111/228, 48.7%), and the average age was 31.5 years, with a high rate of young adults (43.9%, 100/228); 42.5% (97/228) were first-time visitors to the hospital, and 35.5% (81/228) were second-time visitors to the hospital.

Nine of 60 respondents to Part B of the questionnaire were excluded due to careless and repeated answers. These respondents covered all health information departments in Liaoning Province; different position levels may have influenced their opinions about mHealth development (see Table 2).

**Table 1:** Participant demographics of the Part A questionnaire.

Characteristic		n (%)
Total (N)		228 (100.0%)
Age (years)	18-29	100 (43.9%)
	30-40	87 (38.2%)
	41-69	41 (18.0%)
Sex	Male	117 (51.3%)
	Female	111 (48.7%)
Visit type	First	97 (42.5%)
	Subsequent	81 (35.5%)
	Citizen	50 (21.9%)

**Table 2:** Participant characteristics of the Part B questionnaire.

Department	Position			
	Clerk	Chief Information Officer	Engineer	Researcher
Government health information center, n (%)	0 (0.0%)	8 (34.8%)	15 (65.2%)	0 (0.0%)
Hospital, n (%)	8 (57.1%)	1 (7.1%)	1 (7.1%)	4 (28.6%)
Information technology company, n (%)	0 (0.0%)	1 (7.7%)	12 (92.3%)	0 (0.0%)

### Evaluation Outcomes

#### Questionnaire A

Questionnaire A is divided into three parts: *health information-seeking*, *appointment making and medical treatment*, and *smartphone usage*. Comprehensive analyses are detailed in Multimedia Appendix 2.

##### Health Information-Seeking

Regardless of age, sex, or visit type, most of the respondents were unaware of their health conditions (62.3%, 142/228) and unclear about former preventive care records (84.6%, 193/228) or hospital visit records (65.4%, 149/228). Most patients (73.7%, 168/228) were willing to check their health, visit, or test records if it was convenient, with most respondents (64.5%, 147/228) obtaining disease information mostly and/or only from their doctors. Patients (citizens) preferred to get health information from a health expert or hospital visits (65.4%, 149/228) compared to Internet searches (21.1%, 48/228); 55.2% (126/228) indicated that they would likely and/or certainly use the Internet or a mobile app to get some information before seeking a doctor. Additionally, 67.1% (153/228) of respondents answered that they might follow some health suggestions from a prestigious health website; 71.5% (163/228) said they wanted to learn medical-related knowledge if it was convenient. Among all of the above, some significant findings include: women were more likely than men to know partially about their former preventive records (χ^2^=10.855, *P*<0.01); subsequent patients knew more about their health status than first-visit patients (χ^2^=21.075, *P*<0.01); patients were more aware of their former hospital visit records than nonpatients (χ^2^=9.553, *P*<0.05); males were more likely than females to choose Internet searches as the way to obtain health information (χ^2^=10.592, *P*<0.05); first-visit hospital patients would rather choose Internet information-seeking than others (χ^2^=15.612, *P*<0.05); and young adults (18-29 years old) would be more likely than others to use the Internet or a mobile app to obtain some health information before seeking a doctor.

Multiple response analysis showed the most wanted health information included a physical examination, disease awareness, and daily health; computer and smartphone apps were the most favorable ways to search the Internet.

##### Appointment Making and Medical Treatment

Multiple response analysis showed that the three most common problems during medical treatment were long waiting times (66.4%, 148/223), difficulty making appointments (46.5%, 106/228), and unclear treatment results (40.3%, 87/216). Table analysis and Chi square tests showed the most desirable way to make an appointment with a doctor was online (49.6%, 113/228) regardless of age or sex, especially in outpatient departments of internal medicine, otorhinolaryngology, and mental health (χ^2^= 213.077, *P*<0.01). However, the results varied by visit type (χ^2^=25.782, *P*<0.01), which meant citizens were more likely to choose online appointment-making than others. Respondents showed similar degrees of concern regarding online appointment-making, which included: tedious processes (37.3%, 85/228), registering with the wrong department (33.6%, 75/223), inability to find the most wanted doctor (35.0%, 78/223), and more difficult hospital processes (30.9%, 69/223).

Compared with hospital payment (30.7%, 69/225) and uncertain answers (22.7%, 51/225), most respondents indicated that they wanted to pay the appointment-making fee online (46.7%, 105/225) regardless of age, sex, or visit type, especially in outpatient departments of internal medicine, otorhinolaryngology, and mental health (χ^2^= 28.843, *P*<0.05). After online appointment-making, 81.9% (86/105) of the respondents wanted an alert to show the time and process of seeking a doctor in the hospital. If the appointed doctor could not be available on the day (doctors’ schedules are often changed in the hospital), 58.9% (66/112) of respondents chose to make a decision later based on the factual situation, while subsequent visit patients were less likely to continue their hospital visit (χ^2^=10.911, *P*<0.05).

In total, 49.1% (112/228) of participants preferred to choose a time to go to the waiting room and wait for the doctor rather than staying in the waiting room after appointment-making, especially in outpatient departments of internal medicine, otorhinolaryngology, and mental health χ^2^= 27.543, *P*<0.01), regardless of age or visit type. However, females showed more willingness to wait than males (χ^2^=9.968, *P*<0.01). Most respondents only showed partial satisfaction with their medical treatment outcome (54.6%, 112/205). Reasons for dissatisfaction with the doctor-seeking experience included: *still unclear about my condition* (29.1%, 62/213), *have unanswered questions* (49.3%, 105/213), and *doctors’ poor attitude* (21.6%, 46/213).

Thirty-six respondents wrote down other reasons and suggestions about unsatisfactory hospital outpatient experiences, including: five pointed out a lack of clarity of hospital processes and/or ways to choose the correct doctor because of unclear doctor interfaces; three were unclear about the doctors’ prescriptions; three complained about the high medical treatment price, which mainly included medication and physical tests; five respondents did not trust their doctors’ diagnosis; three complained of doctors’ and nurses’ poor service attitudes; five pointed out the prolonged waiting time of outpatients, and waiting for test results after a difficult appointment-making process in the hospital; four respondents were still unclear about all of their conditions or test results; two reported poor navigation and disorder in the outpatient section in the hospital; and four expressed their opinions about the online appointment-making process (Internet problems causing failure, functions that should be customized, mobile payments should also be acceptable, and the process should record the entire view of a patient’s health care).

##### Smartphone and App Usage

We defined a smartphone as a phone that has a standalone operating system, running memory, and connection to wireless Internet and mobile communication Internet (so that users can install any third-party software, such as games, navigation, and so on). Data analysis showed that 96.4% (216/224) of our respondents had smartphones, but department difference (χ^2^=14.18, *P*<0.05) indicated that elderly patients from the ophthalmology department may not use smartphones. Pearson Chi-square test results showed daily time spent on a smartphone significantly declined as the users’ age increased (χ^2^=20.593, *P*<0.01), and differences were noted in different departments (χ^2^=31.613, *P*<0.01). Among the three age groups (18-29, 30-40, >41), the percentages of daily time spent on a smartphone >3 hours were 45.9% (45/98), 28.2% (24/85), and 22.5% (9/40), respectively. Most respondents (69.8%, 150/215) chose “YES” when asked about receiving pushed messages from health and medical-related apps.

Multiple response analysis results showed several trends: the two most used functions of smartphones were apps (85.5%, 189/221) and phone calls (49.3%, 109/221); the two most used kinds of smartphone-based apps were instant messengers (84.2%, 186/221) and news (59.3%, 131/221); the three most desired kinds of apps were health related (63.9%, 138/216), daily life service related (52.3%, 113/216), and medical treatment related (48.6%, 105/216); respondents comprehensively showed high health-related app expectations regarding health management (47%, 101/215), health learning (59.5%, 128/216), and health record queries (52.1%, 112/216); medical-related app expectations included appointment-making (65.1%, 142/218), treatment results queries (59.2%, 129/218), and patient-provider interactions (53.7%, 117/218). The most accepted kinds of push messages from smartphone apps were test results (58.3%, 127/218) and appointment alerts (51.8%, 113/218).

#### Questionnaire B

Crosstab analysis showed that 68% of respondents (34/50) thought mHealth would benefit general citizens more than other institutions, regardless of working units or positions. Multiple response analysis showed: information technology development (71%, 35/49), the health care industry (43%, 21/49), and citizens’ health needs (39%, 19/49) together gave rise to mHealth development; the roles that smartphones play in the mHealth industry mainly involved the information display platform (78%, 39/50) and data collection (or transmission) tools (46%, 23/50); the most used smartphone functions in the health industry were the Internet (70%, 35/50), apps (58%, 29/50), messaging (52%, 26/50), and sensors (42%, 21/50). Based on current health informatization achievements, mHealth was thought to benefit citizens by enhancing health information access (63.3%, 31/49) and improving the hospital visiting process (63.3%, 31/49). Respondents indicated that mHealth usage could benefit the health industry by optimizing health care process management (60%, 30/50) and embodying the value of the whole health informatization structure (48%, 24/50). Data security (49%, 24/49), interoperability (41%, 20/49) and legality (47%, 23/49) were nominated as the main concerns that mHealth is facing in the current situation. The health department (72%, 36/50), hospitals (78%, 39/50), insurance entities (68%, 34/50), researchers (44%, 22/50), and patients (68%, 34/50) were all thought to be important participants in mHealth development. Participants also indicated that data sharing between providers and patients (62%, 31/50), health services (eg, health education and family health; 74%, 37/50) and chronic disease management (56%, 28/50) could be improved by mHealth in the current health environment.

## Discussion

### Principal Results

#### Health Information-Seeking

In this study, we found women were more likely than men to be concerned about health information. Patients were more likely to be familiar with their historical health records than nonpatients, which is in accordance with one of the common Chinese health scenarios, which is that people usually do not care about their health until they get sick. Women are usually more conservative than men regarding new technologies, which explains why males were more likely than females to choose an Internet search as a way to obtain health information. This finding is similar to German research conducted by Bidmon and Terlutter [18], who suggested that women were more engaged in searching for health information, while men were more likely to adapt to a virtual patient-physician relationship, such as personal appointment-making, referral to other doctors, writing prescriptions, discussions of normal test results, and doctor’s notes/certificates of health. First-time hospital-visiting patients would rather choose Internet information-seeking than subsequent patients; young adults (18-29 years old) would be more likely than others to use the Internet or a mobile app to obtain some health information before seeking a doctor, which provides evidence for the conclusion that health communication efforts utilizing mHealth will have a strong impact when the target population is a younger generation [19]. The Internet is increasingly becoming one of the major health information media formats [20]. Patients were favorably disposed to learning health-related information through the Internet or mobile phones. The results above suggest that we should provide functions for checking historical health records and a medical knowledge database for self-help learning when designing an mHealth-based health management platform in Liaoning Province.

#### Hospital Visiting

Significant results include: citizens tended to choose online appointment-making more than first and/or subsequent patients, which is similar to the result for online information-seeking and indicates that people would like to search for health information before seeking a doctor. These responses may be due to limited health care access [21], especially in China. Subsequent patients do not seem to like to continue with hospital visits when their appointed doctor changes, indicating that they want to choose the doctor themselves. This trend indicates another problem in the Chinese health system: patients require a constant primary health care provider instead of seeking unknown hospital doctors [22]. The long waiting time after an outpatient appointment has been made is a major problem during hospital visits; thus, most respondents want to choose an appropriate time to come to the hospital waiting area. However, women are more willing than men to wait until the doctor’s office calls them, which reflects the fact that men in China are more open-thinking and more readily adapt to new things whereas women are more conservative, which is the opposite to the situation in many foreign countries in which women are more likely to seek and accept online health information [18,23].

The findings from this part of the survey have great significance for our platform design and development. In general, we found appointment-making and long waiting times were the most commonly confronted problems in hospital outpatient visiting, and most patients and citizens were willing to make appointments online, which means that designing an online appointment-making function to alleviate the current situation is necessary. However, some patients worried that they might possibly choose the wrong department or doctor, or encounter a more complicated hospital process when making an appointment online, so we need to take these factors into consideration when developing mobile apps. To integrate with the hospital management system, hospitals need to make some changes to the outpatient process to accommodate online appointment-making to make it more convenient. As we perceived, patients would like to pay the appointment fee online, as this is more convenient; after successful payment, most patients want to receive alert messages to remind them of the hospital visiting time, address, and other instructions. Most citizens, and especially patients, would hesitate if the appointed doctor is changed, and when a patient goes to the hospital outpatient department on the appointed day, they do not like to wait for a long time until it is their turn.

According to the results above, we should take every possible expected (or unexpected) circumstance into careful consideration when designing the function frame and workflows of the platform, keeping users fully informed of every change in their appointments. Online payment is a key point for more convenient appointment-making for hospital outpatient visits. However, there are some special challenges that cannot be solved solely by mHealth platform development, such as the increasingly intense relationship between patients and providers, high medical costs and medication prices, and the deficiency of high quality medical resources.

#### Smartphone Usage

The results found in this part are in accordance with current mobile technology development. Almost all respondents had a smartphone, and over half were familiar with smartphone-based apps. Time spent on the Internet on smartphones was negatively correlated with age because most of our respondents were young adults who display online health information-seeking behaviors [20,24]. Most of the respondents would like to try new health apps, such as health management and medical treatment-related apps. In addition to online appointment-making and health history recording, respondents want to use apps to query test results, learn about their conditions, and talk with their doctors. Future research should pay more attention to doctor/patient communication.

#### mHealth Development

We predict the future direction of mHealth is health management and health improvement, targeted at meeting the major requirements of general citizens, such as health management apps, health monitoring, and telemedicine [10,25]. Smartphone apps could be connected with wearable devices through technologies (eg, Bluetooth, Wi-Fi, and wireless sensors) to realize real-time data collection and analytical functions on regional health information platforms, which would provide citizens with online services such as health information storage, information querying, personal health assessments, and chronic disease management [26]. To achieve the final goal of providing more convenient health services for general citizens, mHealth development should be supported by the government, medical institutions, medical insurance, and patients themselves. Governments should set performance standards to evaluate the safety, validity, and practicality, together with hospitals and patients [9,27]. The most common problems we need to solve for mHealth development are safety and interoperability [28]. Due to the explosive increases in health and medical data, data safety is a major problem in the medical field, which is exacerbated by the implementation of mHealth-based app usage [29]. Overall, respondents were all positive about mHealth usage, development, and significance, which is in accordance with current health policy suggestions.

#### General Suggestions for mHealth-Based Health Management Platform Development

Platform development needs to focus more on basic functional designs. Our requirements analysis showed that most citizens were unclear about their historical health records, so the query function design should use this as a foundation. This approach is in accordance with other studies showing that we should provide better means of navigating online health information by improving systems supporting patients’ health information-seeking activities [30,31].

We also need to pay more attention to small details, which is the key to the success of mHealth apps. Study results indicated patients usually encounter many problems during their hospital visits, such as unfamiliarity with hospital outpatient doctors, difficulty knowing which doctor to choose, inability to find a particular location, and frustration from waiting times that are too long during their hospital visits. mHealth app development should try to solve these particular problems.

We also need to address interoperability. Referring to medical treatment functions (eg, appointment making and online payment), hospitals and the mHealth platform should exchange some key parameters, and the hospital should also make some changes to simplify the process of patients visiting their doctors after they have made online appointments, to make it easier for patients to make an online appointment to encourage app usage. Regarding sustainable development, mHealth development needs to continue to devote funds, manpower, and devices to maintain a sustainable operation, which is a key point [32]. Apart from providing free basic services, some customized value-added services are needed and necessary, and more research is needed to investigate what kinds of services would be acceptable.

### Novelty

To our knowledge, this is the first comprehensive study analyzing the requirements of an mHealth platform before proceeding to system design and development in Liaoning Province. We designed and verified a set of questionnaires directed at discovering Internet health information-seeking behavior, online appointment making, mHealth-based app usage, and mHealth development. Based on the current achievements of health informatization, such as the comprehensive regional health platform connecting large hospitals and the unified payment platform, the Health Ministry encourages the development of mHealth-related apps to provide patients with more convenient tools for health management and medical treatment. We believe this study provides many meaningful ideas for functional design and program development of mHealth related apps.

### Limitations

There is the possibility of selection bias among respondents, since we only chose patients (or citizens) who were willing to fill in the questionnaire for us. Young adults showed more interest and understood our questions better, which caused a disproportionate age distribution, and a few elder respondents missed one or two questions, which may be due to their limited health literacy or our incomprehensive design of the questionnaire. There may be more health information-seeking problems or requirements that will need to be explored in the future. It is very common in China that elders use fewer computer or smartphone functions than young adults, even when they have access to the Internet, and we also saw this trend in our results. We believe that the design of the mHealth app should mainly be oriented to young adults, who will help elder family members when seeking a doctor in a hospital online, and will also help elder people improve their online health information-seeking behavior [33]. Furthermore, our study is simply an analysis of the requirements for a large-scale information system development process; the effectiveness of our study should be evaluated according to the implementation of the mHealth-based platform and other similar projects.

## Appendix

Multimedia Appendix 1 mHealth requirement survey.

Multimedia Appendix 2 Health information seeking characteristics.

## Abbreviations

IT: information technology
mHealth: mobile health
