# Supplementary material for: Analysis of Requirements for Developing an mHealth-Based Health Management Platform
Source: JMIR Mhealth Uhealth. 2017 Aug 3;5(8):e117. doi: 10.2196/mhealth.5890 (PMC5561389; doi:10.2196/mhealth.5890)
Supplement: Multimedia Appendix 1 [file mhealth_v5i8e117_app1.pdf]

Appendix1

mHealth requirement survey(part A)

Hello, my admired friend! I am a graduate student from China Medical University. As the development of current health informatization as well as popularity of Internet and smartphone, simple and convenient phone based applications (APPs) can help you solve many health and medical related problems, thus we need to know about your daily behaviors and problems during your daily healthcare and medical seeking, after further analysis, we will know how to better utilize mobile information technology to solve your problems specially

As a part of health management program in Liaoning Province , this survey is proposed by China Medical University, the results are confidential and will only be used for research . Thanks for your cooperation! (questions in italics are multiple choices)

Questionnaire source：China Medical University

Age period： □18-29 □30-40 □>=41

Sex: □male □female

Visit type： □First □Subsequent

Health and Medical：

Q1: Are you clear about your health condition?

1.No 2.Uncertain 3.Yes

Q2: Are you clear about your former prevention care records?

1. Not at all 2.partially 3.totally

Q3: Are you clear about your former hospital visit records?

1. Not at all 2.partially 3.totally

Q4: Are you willing to check your health, visit or test records if it is very convenient?

1.No 2.Maybe 3.Yes

Q5:Which kind of history records do you want to check at any time?

1.Physical examination 2. Follow-up of chronic disease 3. Newborn prevention care 4. Hospital visit 5. Maternal health care 6. Health management of the aged 7. Health education

Q6: Do you get your disease information mainly from your doctor?

1. No 2.Some 3.Much 4.Totally

Q7: Which way do you prefer to know about your condition？

1. Check former records 2.experts consultation 3.Internet search 4.Hospital visits

Q8: Would you like to get some information from the Internet or mobile APP before seeking a doctor？

1. No 2.Some 3.Much 4.Totally

Q9: If you can get some health suggestions from a medically approved health website, will you follow them ？

1. No 2.Some 3.Much 4.Totally

Q10: Will you learn related medical knowledge voluntary if it's

convenient?

1.No 2.Maybe 3.Yes

Q11:what medical knowledge do you want to learn most？

1.Disease awareness 2.Medicine awareness 3.Test&examination 4.Daily healthcare 5.Maternal care knowledge

Q12: what kinds of tools do you want to use to check health information online?

1. Smartphone app 2.tablets 3.Computer 4.Paper

Q13: what barriers do you meet during your medical seeking process?

1. Appointment registration difficulty 2.Long waiting time 3.Communication disorders with doctor 4.Unclear of treatment results

Q14: Which way do you like best to have an appointment registration?

1. Hospital 2.Phone call 3.Online

Q15:what problems are you worry about when making an appointment online?

Tedious process 2.Register a wrong department 3.Cannot find the most wanted doctor 4.More difficult in hospital process

Q16: Do you want to pay the registration fee online or in the hospital?

1.Hospital 2.Uncertain 3.Online

Q17:what alerts do you want to receive after online appointment making□

1SMS alert of time& process 2Docotor change 3Others

Q18: If your appointed doctor cannot be available, will you continue to see another doctor or apply for refund?

1. Continue 2.Refund 3.Make decision later

Q19: Would you like to stay in the waiting room or choose a right time?

1. wait 2.choose a right time 3.that depends

Q20: Are you satisfied with your treatment results□

1.No 2.Partially 3.satisfied 4.quite satisfied

Q21:Resons for your unsatisfactory□

1Still unclear about my condition 2Have unanswered questions 3The doctor is not nice to me

Q22: Other problem in your hospital visit or daily healthcare□

____

Smartphone□

Introduction□ Refers to one kind of mobile phone that have independent operating system, users can install t navigation software, games, applications provided by third-party service , realize the wireless Internet access through the mobile communication network . Brands like Apple, Samsung are world popular, Lenovo, XIAOMI are common in China.

Q1:Do you have a Smartphone？

1.No 2.Yes

Q2:How many time do you usually spend on your Smartphone？

1. <= 1h 2.1h-2h 3.3h-5h 4.>=6h

Q3:what functions of Smartphone do you most often used in your daily life？

1.phone call 2.SMS 3.APPs（such as Wechat） 4Webpage

Q4:what APPs do you most often used？

1.Maps 2.News 3.Instant messaging 4.Games

Q5:what APPs are you expecting to you in future?

1.Health related 2.treatment related 3.hobbies related 4.daily services

Q6:what health related APPs are you expecting？

1.health management 2. Health knowledge 3.information search

Q7:what treatment related APPs are you expecting?

1.online appointment making 2. Treatment results query 3patients and providers interaction 4.self-help triage

Q8: do you want to see the services hospital pushed service messages about health and treatment by Smartphone?

1.No 2.Uncertain 3.Yes

Q9: what kind of pushed services do you want to receive by Smartphone APPs?

1Appointment alert 2.test results 3.health message4.Daily

healthcare5.Hospital updates
