# Supplementary material for: Analysis of Requirements for Developing an mHealth-Based Health Management Platform
Source: JMIR Mhealth Uhealth. 2017 Aug 3;5(8):e117. doi: 10.2196/mhealth.5890 (PMC5561389; doi:10.2196/mhealth.5890)
Supplement: Multimedia Appendix 2 [file mhealth_v5i8e117_app2.pdf]

comprehensive analysis		Age				Sex			Visit type (patients&citizens)				medicine speciality						
		18~29	30~40	41-69	Chi-square	male	female	Chi-square	first	subsequent	citizen	Chi-square	medicine	dermatology	gynecology	ophthalmology	otorhinolaryngology	mental health	Chi-square
		count	count	count	P value	count	count	P value	count	count	count	P value	coun	count	count	count	count	count	P value
Would you like to stay in the waitting room or choose a right time?	total count	98	85	41		114	110		95	80	49		47	33	31	40	48	24	
	stay	17	13	10	0.734	12	28	0.007**	21	13	6	0.191	6	7	4	17	4	2	0.002**
	choose a right time	50	43	17		65	45		42	37	31		28	11	15	14	28	13	
	that depends	31	29	14		37	37		32	30	12		13	15	12	9	16	9	
Are you satisfied with your treatment resutls?	total count	98	85	41		114	110		95	80	49		47	33	31	40	48	24	
	no	5	5	1		6	5		6	2	3		4	0	0	3	3	1	
	partially	44	51	18	0.304	55	58	0.054	54	40	19	0.057	17	18	22	18	25	13	0.195
	quite	42	26	20		51	37		27	35	26		25	13	6	16	19	8	
	yes	7	3	2		2	10		8	3	1		1	2	3	3	1	2	
smartphonte own	total count	98	85	41		114	110		95	80	49		47	33	31	40	48	24	
	no	1	3	4	0.041*	3	5	0.440	5	2	1	0.499	1	0	2	5	0	0	0.015*
	yes	97	82	37		111	105		90	78	48		46	33	29	35	48	24	
time spent on smartphone	total count	98	85	40		114	109		95	79	49		47	33	30	40	48	24	
	<1hours	5	11	5		11	10		9	8	4		4	2	3	11	1	0	
	1~2hours	35	42	26	0.002**	52	51	0.841	35	40	28	0.215	17	18	16	17	23	12	0.007**
	3~5hours	45	24	9		42	36		38	25	15		21	9	6	11	21	9	
	>6hours	13	8	0		9	12		13	6	2		5	4	5	1	3	3	
receive pushed message	total count	98	82	40		113	107		93	78	49		47	32	30	39	48	23	
	no	8	5	2	0.268	9	6	0.657	2	7	6	0.108	2	0	2	6	3	1	0.149
	uncertainty	28	22	5		26	29		28	18	9		13	7	8	13	12	2	
	yes	62	55	33		78	72		63	53	34		32	25	20	20	33	20	
Which way do you like best to have an appointment&registration?	total count	100	85	41		116	110		96	81	49		47	34	32	40	48	24	
	hosptal	27	28	14	0.231	35	34	0.058	35	30	4	0.000**	4	17	14	33	0	0	0.000**
	phone call	15	18	11		16	28		20	19	5		1	17	16	7	3	0	
	online	58	39	16		65	48		41	32	40		42	0	2	0	45	24	
Do you want to pay the registration fee online or in the	total count	99	85	41		115	110		95	81	49		47	34	31	40	48	24	
	hospital	34	25	10	0.482	29	40	0.149	33	24	12	0.192	11	12	9	18	13	5	0.001**
	uncertainty	18	20	13		26	25		23	21	7		7	13	11	11	5	4	
	online	47	40	18		60	45		39	36	30		29	9	11	11	30	15	
